# Extravesical vs. intravesical ureteric reimplantation for primary vesicoureteral reflux: A systematic review and meta-analysis

**DOI:** 10.3389/fped.2022.935082

**Published:** 2022-10-21

**Authors:** Zhi Wei Law, Caroline C. P. Ong, Te-Lu Yap, Amos H. P. Loh, Udayan Joseph, Siam Wee Sim, Lin Yin Ong, Yee Low, Anette S. Jacobsen, Yong Chen

**Affiliations:** ^1^Department of Urology, Singapore General Hospital, Singapore, Singapore; ^2^Department of Pediatric Surgery, KK Women’s and Children’s Hospital, Singapore, Singapore

**Keywords:** vesicoureteral reflux (VUR), reimplantation, extravesical, intravesical, recurrence

## Abstract

**Purpose:**

This study aims to compare the outcomes of extravesical (EVUR) and intravesical (IVUR) ureteric reimplantation for primary vesicoureteral reflux (VUR) *via* systematic review and meta-analysis.

**Methods:**

Literature review from Medline, Embase, and Cochrane since inception to March 2022 was performed. Meta-analysis was conducted on eligible randomized controlled trials (RCT) and observational cohort studies (OCS) comparing outcomes between EVUR and IVUR.

**Results:**

Twelve studies were included, comprising 577 patients (778 ureters) operated by EVUR and 395 patients (635 ureters) by IVUR. Pre-operative VUR grade, postoperative VUR persistence and hydronephrosis was not statistically significant. EVUR had shorter operative time [mean differences (MD) −22.91 min; 95% confidence interval (CI), −44.53 to −1.30, *P* = 0.04] and hospital stay (MD −2.09 days; 95% CI, −2.82 to −1.36, *P* < 0.00001) compared to IVUR. Bilateral EVUR had higher risk of postoperative acute urinary retention (ARU) (8.1%) compared to bilateral IVUR (1.7%) (OR = 4.40; 95% CI, 1.33–14.58, *P* = 0.02). No patient undergoing unilateral EVUR or IVUR experienced ARU.

**Conclusion:**

Both EVUR and IVUR are equally effective in correcting primary VUR. Operative time and hospital stay are shorter after EVUR compared to IVUR. However, bilateral EVUR is associated with higher risk of postoperative ARU.

## Introduction

Vesicoureteral reflux (VUR) is a prevalent problem afflicting 1% of the pediatric population (1). Primary VUR is found in 30–45% of the children with febrile urinary tract infection (UTI) (2) and increases the risk of recurrent febrile UTI and renal scarring (3). Untreated recurrent UTI complicated by reflux nephropathy is associated with secondary hypertension and end-stage renal disease afflicting 10–20% of these children (4). Spontaneous resolution of lower grade VUR is more common with resolution rates of 80% for VUR grades I and II vs. 30–50% for VUR grades III–V (5). Surgical intervention to preserve renal function is indicated in children with persistent VUR, breakthrough UTI or have interval progression of renal scarring (6).

Surgical options for correction of VUR include ureteral reimplantation and endoscopic injection of bulking agents. Increased adoption of endoscopic injection has resulted in decline in open surgery (7), nevertheless, ureteral reimplantation remains relevant and is the gold standard for surgical treatment of persistent high grade VUR, especially if symptomatic, or when VUR is associated with paraureteral Hutch’s diverticulum. Ureteral reimplantation can be conducted *via* intravesical and extravesical techniques; both approaches have reported low complication rates and excellent success rates (92–98%) (8). Cohen cross-trigonal reimplantation is the most popular intravesical ureteral reimplantation (IVUR) technique as it is easy to teach and replicable. However, potential risks with cystotomy and the cross-trigonal submucosal tunneling technique include postoperative hematuria, more postoperative pain, longer length of hospital stay (LOS), longer duration of urinary catheterization and technical difficulty for future upper tract endoscopy. In contrast, extravesical ureteral reimplantation (EVUR) approaches do not require cystotomy to achieve surgical correction so are potentially less invasive with shorter LOS and shorter operative times compared to IVUR techniques. An extravesical approach is preferred when contemplating minimally invasive surgery (MIS), since MIS EVUR utilizes the familiar laparoscopic technique that is technically less challenging than transvesical MIS IVUR (9). However, EVUR involves extravesical dissection near the distal ureter that may compromise detrusor innervation at the trigone. This potentially increases the risk of postoperative acute retention of urine (ARU) with need for transient or prolonged urinary catheterization, particularly for bilateral EVUR (10).

Several underpowered studies have been published that compare the outcomes of EVUR and IVUR but there has been no consensus regarding which approach is better, thus we reviewed the literature to conduct a meta-analytic review. To the best of our knowledge, this is the first systematic review and meta-analysis comparing the operative outcomes of EVUR and IVUR for pediatric primary VUR.

## Materials and methods

### Study selection

The systematic review was conducted following the Preferred Reporting Items for Systematic Reviews and Meta-Analyses (PRISMA) guidelines. An electronic search from databases of PubMed, Embase, and Cochrane Library was performed by two independent authors (ZL and UJ) from the date of database inception until March 2022. The following terms were applied using different permutations: “ureter* reimplant*,” “ureteroneocystostomy,” “pediatric OR child OR children,” “vesicoureteral reflux OR vesicoureteric reflux,” “ureter reflux OR ureteral reflux OR ureteric reflux,” “extravesical,” and “intravesical.” A secondary search of the references from the retrieved articles was performed to identify other potentially eligible studies. Due to lack of randomized controlled trials (RCT), observational cohort studies (OCS) were also included. All articles included in this meta-analysis were published in English, although no language restriction was imposed during the initial literature search.

### Data extraction

Two independent reviewers (CY and ZL) evaluated the selected studies, extracted and tabulated the data from each article – first author, year of publication, study design, sample size, follow-up period, mean age at operation, preoperative VUR grade and clinical outcomes including postoperative VUR persistence, ureteric obstruction/hydronephrosis, ARU, duration of operation and length of stay (LOS). Any discrepancies were resolved *via* open discussion till both reviewers reached consensus at each stage of the process.

### Inclusion criteria

We included all studies published as a full indexed journal article that compared the outcomes of EVUR vs. IVUR for primary VUR in children.

### Exclusion criteria

The following were excluded from this meta-analysis: studies that included patients with ectopic ureter, ureterocele, posterior urethral valve, neurogenic bladder, and/or previous bladder surgery; studies that did not report outcomes separately for IVUR and EVUR; studies with data that overlapped with earlier reports.

### Subgroup analysis

Subgroup analysis was performed in comparison of EVUR and IVUR in open or minimally invasive surgery (MIS) separately. The outcome of EVUR and IVUR were also compared in a subgroup of patients with bilateral VUR.

### Assessment of methodological quality of included studies

The Newcastle-Ottawa scale (total 9 stars) was used to assess the methodological quality for the OCS where low risk of bias is allocated ≥7 stars, moderate risk with 4–6 stars, and high risk with ≤3 stars. The RCT was evaluated using the Jadad score (total 5 points) where scores above 3 are considered high quality.

### Statistical analysis

Pooled odds ratios (OR) were calculated for dichotomous variables using the Mantel-Haenszel method. Pooled mean differences (MD) were measured for continuous variables using the inverse variance method in the meta-analysis. The confidence interval (CI) was established at 95% and *P*-values of less than or equal to 0.05 were considered statistically significant.

Statistical heterogeneity was assessed using *I*^2^. A fixed effects model was used if *I*^2^ < 25% and a random effects model was used if *I*^2^ ≥ 25%.

## Results

### Study characteristics

**FIGURE 1:**
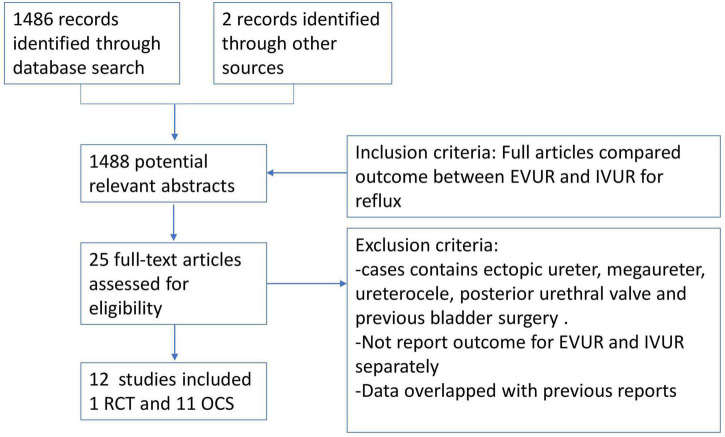
Preferred Reporting Items for Systematic Reviews and Meta-Analyses (PRISMA) flowchart of selection of articles.

A total of 1,488 studies were identified from the literature search. Twenty-five studies fit inclusion criteria but 13 were removed because of meeting the exclusion criteria. In the end, 12 studies comprising 1 RCT and 11 OCS (1 prospective and 10 retrospective) were eligible for meta-analysis (Figure 1) (11–22).

**FIGURE 2:**
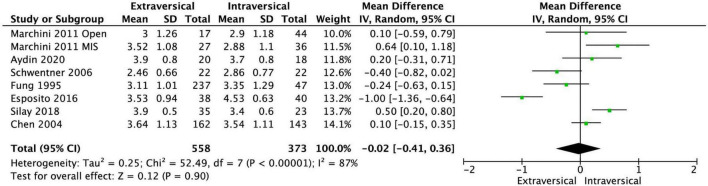
Forest plot of preoperative VUR grade in EVUR vs. IVUR group.

**FIGURE 3:**
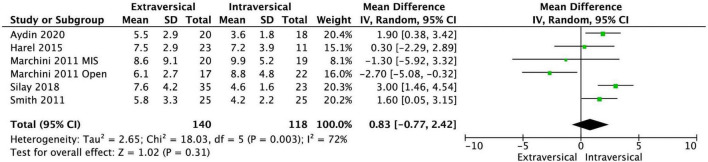
Forest plot of age at operation in EVUR vs. IVUR group.

The baseline characteristics of the twelve included studies are shown in Table 1. Our meta-analysis included a total of 972 patients with VUR in 1,413 ureters. The EVUR group had 577 patients (778 ureters) and the IVUR group had 395 patients (635 ureters). There was no statistical difference in preoperative VUR grade (MD = −0.02; 95% CI, −0.41 to 0.36; *P* = 0.90; *I*^2^ = 87%) and age at operative (MD = 0.83; 95% CI, −0.77 to 2.42; *P* = 0.31; *I*^2^ = 72%) between EVUR and IVUR groups (Figures 2, 3).

**TABLE 1:** Characteristics and outcome of studies included in the meta-analysis.

Study (Author, year)	Study type	No. of patient (No. of Ureters)	Operative methods	Age at operation (years)	Preoperative reflux grade (mean ± SD)	Operative time (minutes)	Length of stay (days)
Ellsworth 22	OCS	Extra: 29 (38)	Extra: Open Destrusorrhaphy	NA	NA	NA	Extra: 2.7** ±** 1.03
	(retrospective)	Intra: 27 (43)	Intra: Open PL				Intra: 4.4** ±** 1.8
Fung 11	OCS	Extra: 161 (237)	Extra: Open Destrusorrhaphy	Extra: 6.1	Extra: 3.11** ±** 1.01	NA	NA
	(retrospective)	Intra: 27 (47)	Intra: Open Paquin	Intra: 5.5	Intra: 3.35** ±** 1.29		
Chen 12	OCS	Extra: 126 (162)	Extra: OpenvDestrusorrhaphy	3.5** ±** 2.7 (0.7-12.5)	Extra: 3.64** ±** 1.13	Unilateral / Bilateral	Unilateral / Bilateral
	(retrospective)	Intra: 92 (143)	Intra: Open Cohen		Intra: 3.54** ±** 1.11	Extra : 58** ±** 12 / 94** ±** 24	Extra Open: 3.0** ±** 1/2.8** ±** 1.2
						Intra: 139** ±** 13 / 181** ±** 17	Intra: 4.9** ±** 1 / 5.5** ±** 1.7
McMann 13	OCS	Extra: 18 (33)	Extra: Open Destrusorrhaphy	8.56 (0.9-23)	NA	NA	Extra: 3.00** ±** 1.33
	(retrospective)	Intra: 12 (22)	Intra: Open Cohen				Intra: 5.36** ±** 1.75
Schwentner 14	RCT (Prospective)	Extra: 22	Extra: Open LG	Extra: 5.8 (1.5-9.2)	Extra: 2.46** ±** 0.66	Extra: 66.73** ±** 9.68	Extra: 3.7** ±** 0.5
		Intra: 22	Intra: Open PL	Intra: 5.2 (0.7-9.2)	Intra: 2.86** ±** 0.77	Intra : 79.24** ±** 6.9	Intra: 4** ±** 0.3
Marchini 15	OCS	Extra: 37 (44)	Extra: Open (17)	Extra Open: 6.1** ±** 2.7	Extra Open: 3.00** ±** 1.26	Extra Open: 120** ±** 47.5	Extra Open: 1.7** ±** 1.0
	(retrospective)	Intra: 41 (82)	Robot LG (20)	Extra Robot: 8.6** ±** 9.1	Extra Robot: 3.52** ±** 1.08	Extra Robot: 233.5** ±** 60.2	Extra Robot: 1.7** ±** 1.0
			Intra: Open GA/cohen (22)	Intra Open: 8.8** ±** 4.8	Intra Open: 2.90** ±** 1.18	Intra Open: 147.5** ±** 34.3	Intra Open: 2.9** ±** 1.0
			Robot GA/cohen (19)	Intra Robot: 9.9** ±** 5.2	Intra Robot: 2.88** ±** 1.10	Intra Robot: 232.6** ±** 37.4	Intra Robot: 1.8** ±** 1.2
Smith 16	OCS	Extra: 25 (33)	Extra: Robot LG	Extra Robot: 5.8** ±** 3.3	NA	Extra: 185** ±** 41.6	Extra: 1.4** ±** 0.5
	(retrospective-matched)	Intra: 25 (46)	Intra: Open Cohen	Cohen: 4.2** ±** 2.2		Intra: 165** ±** 23.1	Intra : 2.1** ±** 0.7
Harel 17	OCS (Prospective)	Extra: 23	Extra: Robot	Extra robot: 7.5** ±** 2.9	NA	Extra robot: 204** ±** 34	Extra: 1 day 20/23 pts (87%), = 2 days 3/23 pts (13%)
		Intra: 11	Intra: Open PL/cohen/GA	Intra open: 7.2** ±** 3.9		Intra open: 188** ±** 36	Intra : 1 day 6/11 pts (55%), = 2 days 5/11 pts (45%)
Esposito 18	OCS	Extra: 30 (38)	Extra: Lap LG	4.86 (0.9-12)	Extra: 3.53** ±** 0.94	Extra UL: 95.50** ±** 33.59	Extra : 2.41 ** ±** 0.867
	(retrospective)	Intra: 30 (40)	Intra: Open cohen		Intra: 4.53** ±** 0.63	Extra BL: 128.60** ±** 36.58	Intra : 12.58** ±** 4.263
						Intra UL: 109.35** ±** 17.73	
						Intra BL: 149.15** ±** 28.75	
Sriram 19	OCS	Extra: 51 (83)	Extra: Open LG	Extra: 1.3 (0.8-2)	NA	Extra UL: 62** ±** 21	Extra UL: 4.2** ±** 1.4
	(retrospective)	Intra: 67(134)	Intra: Open cohen	Intra: 3 (2-5)		Extra BL: 104** ±** 18	Extra BL: 4.6** ±** 1.6
						Intra BL: 128** ±** 15	Intra BL: 6.5** ±** 0.5
Silay 20	OCS	Extra: 35	Extra: Open LG	Extra: 7.6** ±** 4.2	Extra: 3.9** ±** 0.5 (3-5)	Extra: 87** ±** 29.8	Extra: 1.2** ±** 0.6
	(retrospective)	Intra: 23	Intra: Open Cohen	Intra: 4.6 ± 1.6	Intra: 3.4** ±** 0.6 (3-5)	Intra: 110.3** ±** 16.9	Intra: 2.8** ±** 0.8
Aydin 21	OCS	Extra: 20	Extra: Open LG	Extra: 5.5** ±** 2.9	Extra: 3.9** ±** 0.8	Extra: 72.6** ±** 10.4	Extra: 2.0** ±** 0.4
	(retrospective)	Intra: 18	Intra: Open Cohen	Intra: 3.6** ±** 1.8	Intra: 3.7** ±** 0.8	Intra: 85** ±** 11.4	Intra: 4.6** ±** 0.9
Ellsworth 22	OCS	Extra: 2/38 (5.3%)	Extra: 2 (Bilatral)	Extra: 1			NA	US 1.5m,6m VCUG 6m
	(retrospective)	Intra: 2/43(4.7%)	Intra: 0	Intra: 2				
Fung 11	OCS	Extra unilateral: 0/84	Extra: 3 (Bliateral)	NA			NA	NA
	(retrospective)	Extra bilateral: 3/146 (2.1%)	Intra: 0					
		Extra total: 3/230 (1.3%)						
		Intra: 1/47 (2.1%)						
Chen 12	OCS	Extra : 1/162(0.6%)	Extra: 4	Extra : 3			57.6 (30-96)	NA
	(retrospective)	Cohen: 1/143 (0.7%)	Intra: 3 (IDC clotted)	Intra: 1				
McMann 13	OCS	Extra: 2/33	Extra: 0	Extra: 1			NA	US 1.5w
	(retrospective)	Intra: 0/22	Intra: 0	Intra: 2				VCUG 3 m
Schwentner 14	RCT (Prospective)	Extra: 0	NA	Extra: 4	Extra: 0		32 (15-42)	US, 3, 6 m VCUG 6 m
		Intra: 0		Intra: 1	Intra: 4.2** ±** 1.2			
Marchini 15	OCS	Extra Open: 1/17 (5.9%)	Extra Open: 0	0			Extra Open: 12.8** ±** 7.5	US 1,3,12,24m RNC 3m
	(retrospective)	Extra Robot: 0/27 (0%)	Extra Robot: 2 (Bilatral)				Extra Robot: 12** ±** 14.3	
		Intra Open: 3/44 (6.8%)	Intra Open: 0				Intra Open: 12.1** ±** 10.8	
		Intra Robot: 3/38 (7.9%)	Intra Robot: 1 (Bilatral)				Intra Robot: 19.4** ±** 18.2	
Smith 16	OCS	Extra: 1/31 (3.2%)	Extra: 3 (Bilatral)	Extra: 1			Extra: 16.0 (2-44)	US 1 m
	(retrospective-matched)	Intra: 0/46 (0%)	Intra : 0	Intra: 2			Intra: 29.1 (6-41)	VCUG 3-4m
Harel 17	OCS (Prospective)	NA	NA	NA			NA	NA
Esposito 18	OCS	Extra: 4/38 (10.5%)	Extra: 0	Extra: 0	Extra: 0	Extra: 0	Extra : 34.8	US 6m
	(retrospective)	Intra: 5/40 (12.5%)	Intra : 1	Intra: 1	Intra: 28	Intra: 30	Intra: 37.2	VCUG 6m
Sriram 19	OCS	Extra : 4/83 (4.8%)	NA	NA	Extra:10		36 (12-84)	NA
	(retrospective)	Intra : 5/134 (3.7%)			Intra: 31			
Silay 20	OCS	Extra: 2/35 (5.7%)	NA	NA			Extra: 21** ±** 14.7	VCUG 6-12m
	(retrospective)	Intra: 0/23 (0%)					Intra: 13.1** ±** 10.4	
Aydin 21	OCS	Extra: 0/20 (0%)	NA	NA	Extra: 0		12	VCUG 6 m
	(retrospective)	Intra: 0/18 (0%)			Intra: 2.5** ±** 1.3			

RCT, randomized controlled trials; OCS, observational clinical studies; UL, unilateral, BL, bilateral, NA, not applicable; PL, Politano-Leadbetter; GA, Glen-Anderson advancement; LG, Lich-Gregoir; MIS, minimally invasive surgery; Lap, laparoscopic; US, ultrasound; VCUG, voiding cystourethrography; RNC, radionuclide cystography.

All studies had a follow up period of at least 1 year, and the study mean follow up duration ranged from 12 to 57.6 months. The age the patients, in the included study, ranged from 0.8 to 23 years of age. Eleven studies were completely pediatric studies, while 1 study McMann 2004 included patients above 17 years of age. We chose to include McMann 2004 as majority of the patients were pediatric with a mean age of 8.5 years of age. For the EVUR group, there were 479 open EVUR and 98 MIS EVUR surgeries. For the IVUR group, there were 376 open IVUR and 19 MIS IVUR surgeries. The various operative techniques employed are detailed in Table 1. Common outcome measures that could be evaluated from the studies included: persistent postoperative reflux, postoperative ARU, operative time, and length of stay.

### Methodological quality of included studies

Of the eleven OCS, four studies have moderate risk of bias (score <7) and the remaining seven studies are at low risk (score ≥7). The sole RCT in the meta-analysis was rated low quality with Jadad score of 1 star (Table 2).

**TABLE 2:** Assessment of methodological quality of included studies.

The Newcastle-ottawa scale for assessment of cohort studies									
**Studies**	**Selection**				**Comparability**	**Outcome**			**Overall score**
	**Representativeness of the exposed cohort**	**Selection of the non exposed cohort**	**Ascertainment of exposure**	**Demonstration that outcome of interest was not present at start of study**	**Comparability of cohorts on the basis of the design or analysis**	**Assessment of outcome**	**Was follow-up long enough for outcomes to occur**	**Adequacy of follow up of cohorts**	
Ellsworth 22	*		*	*		*		*	5
Fung 11	*	*	*	*	**			*	7
Chen 12	*	*	*	*	**	*	*	*	9
McMann 13	*	*	*	*		*		*	6
Marchini 15	*		*	*	*	*	*	*	7
Smith 16			*	*	*	*	*	*	6
Harel 17	*	*	*	*	*	*		*	7
Esposito 18	*	*	*	*	**	*	*	*	9
Sriram 19	*		*	*		*	*	*	6
Silay 20	*	*	*	*	**	*	*	*	9
Aydin 21	*	*	*	*	**	*	*	*	9

**The Jadad score for assessment of randomized control trials**									

**Studies**		**Randamization (2 points)**		**Blinding (2 points)**		**Withdraws (1 points)**		**Overall score**	

		1 point if radomization is mentioned; another point if method of radomization is appropriate; deduct 1 point if method of radomenization is inappropriate		1 point if blinding is mentioned; another point if method of blinding is appropriate; deduct 1 point if method of blinding is inappropriate		1 point if withdraw/dropout is mentioned;			
Schwentner 2006		1 points		0 point		0 point		**1**	

### Persistent vesicoureteral reflux after operation

**FIGURE 4:**
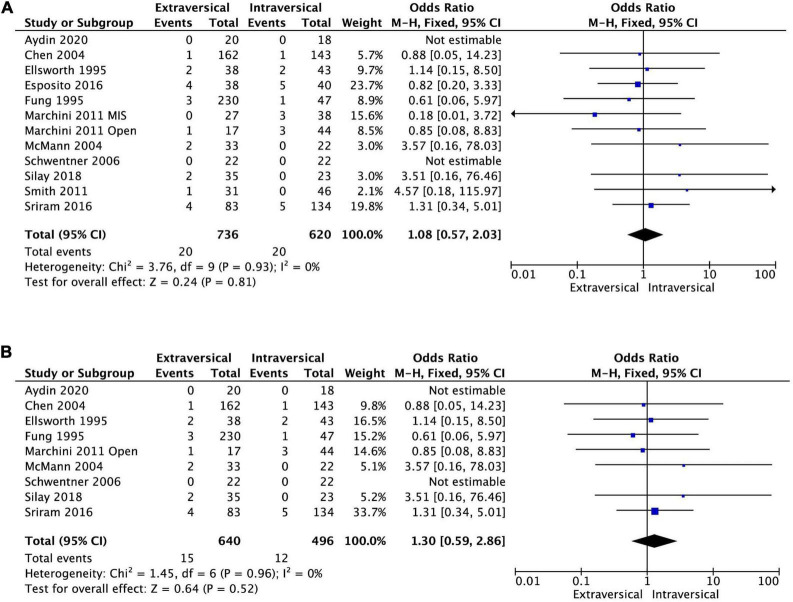
Forest plot of persistence of reflux after **(A)** EVUR vs. IVUR in general, and after **(B)** open EVUR vs. open IVUR.

Eleven studies reported outcomes of ureteric reimplantation in terms of the persistence of VUR after operation. There was no statistically significant difference in the incidence of persistent VUR after surgery between EVUR (20 out of 736 ureters, 2.8%) and IVUR (20 out of 620 ureters, 3.3%) groups. The pooled OR was 1.08 (95% CI, 0.57–2.03, *P* = 0.81, *I*^2^ = 0%) (Figure 4A).

### Postoperative hydronephrosis

**FIGURE 5:**
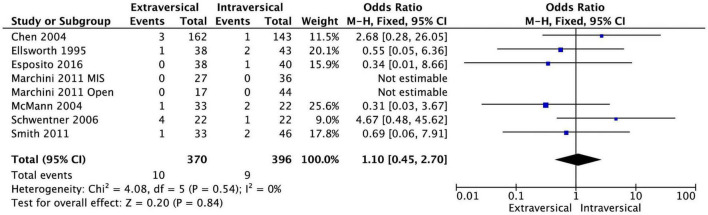
Forest plot of postoperative hydronephrosis after EVUR vs. IVUR for primary VUR disease.

Rates of postoperative hydronephrosis were not statistically significant between EVUR (10 out of 370 kidneys, 2.7%) and IVUR (9 out of 396 kidneys, 2.3%) based on seven studies, where this data was available. The pooled OR was 1.10 (95% CI, 0.45–2.70, *P* = 0.84, *I*^2^ = 0%) (Figure 5).

### Postoperative acute retention of urine

**FIGURE 6:**
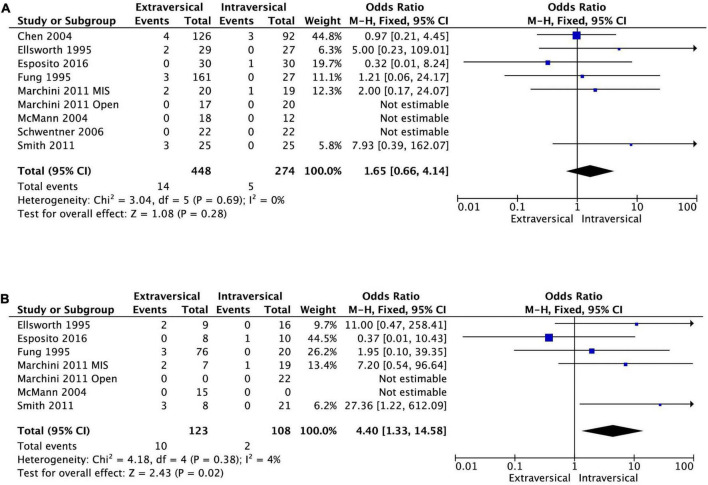
Forest plot of acute retention of urine after EVUR vs. IVUR for VUR in general **(A)** and in bilateral VUR disease **(B)**.

Eight studies had sufficient data for the meta-analysis of the incidence of postoperative ARU. There was no statistically significant difference in the odds of having ARU after EVUR (3.1%) and IVUR (1.8%), the pooled OR was 1.65 (95% CI, 0.66–4.14, *P* = 0.28, *I*^2^ = 0%) (Figure 6A). However, bilateral EVUR was associated with significantly higher postoperative ARU compared to bilateral IVUR as shown in the following subgroup analysis (Figure 6B). Two studies did not specify if ARU was related to unilateral or bilateral surgery; the rest of the studies did not report any patient developing ARU after unilateral reimplantation.

### Operative time per patient

**FIGURE 7:**
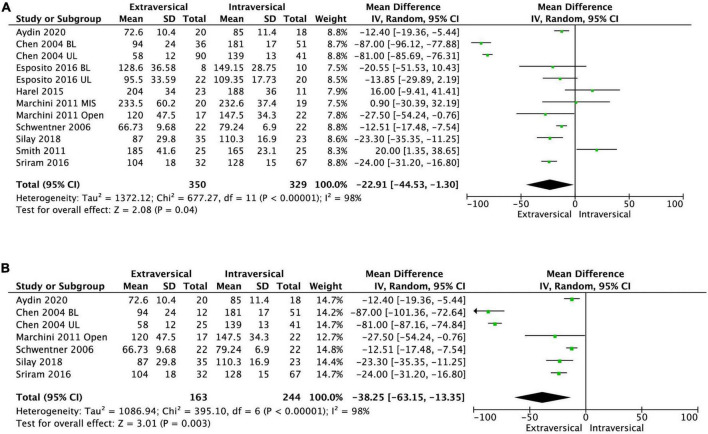
Forest plot of operative time after **(A)** EVUR vs. IVUR for VUR in general and **(B)** open EVUR vs. open IVUR.

Nine studies had analyzable data on the duration of surgery. EVUR had significantly shorter operative time compared to IVUR; MD was −22.91 min (95% CI, −44.53 to −1.30, *P* = 0.04, *I*^2^ = 98%) (Figure 7A).

### Length of stay

**FIGURE 8:**
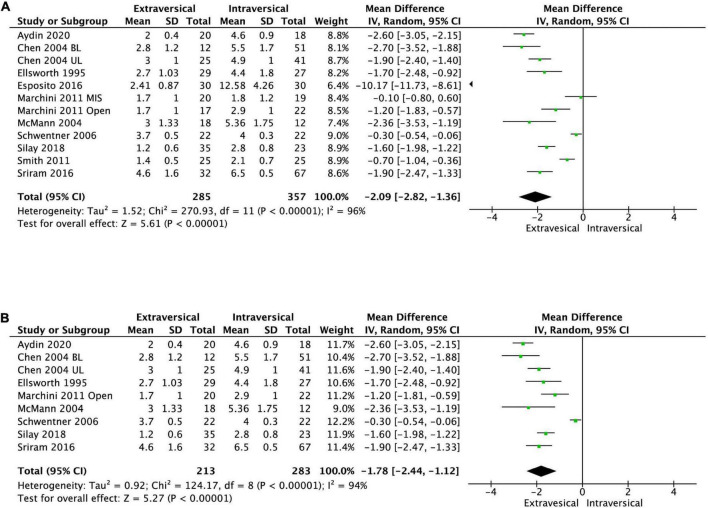
Forest plot of length of stay after **(A)** EVUR vs. IVUR for VUR in general and **(B)** open EVUR vs. open IVUR.

Ten studies reported the LOS after ureteric reimplantation. EVUR patients had significantly shorter LOS compared to those who underwent IVUR (MD −2.09 days; 95% CI, −2.82 to −1.36, *P* < 0.00001, *I*^2^ = 96%) (Figure 8A).

### Duration of urinary catheterization

Information about duration of urinary catheterization was not routinely reported, but when reported, was shorter duration in the EVUR group (14, 15, 20, 21). However, the included studies did not provide mean and SD on duration of urinary catheterization for meta-analysis.

### Subgroup analysis

#### Extravesical ureteral reimplantation vs. intravesical ureteral reimplantation with open approach

There was no significant difference in the persistent postoperative VUR between open EVUR and open IVUR (pooled OR of 1.3; 95% CI, 0.59–2.86, *P* = 0.52, *I*^2^ = 0%, Figure 4B). However, the operative time for open EVUR was significantly shorter compered to open IVUR for combined unilateral and bilateral reflux (MD −38.25 min; 95% CI, −63.15 to −13.35; *P* = 0.003, *I*^2^ = 98%, Figure 7B), as well as for unilateral reflux (MD −14.01 min; 95% CI, −18.89 to −9.14; *P* < 0.0001, *I*^2^ = 23%). Open EVUR patients also had significantly shorter LOS compared to open IVUR with a MD of −1.78 days (95% CI, −2.44 to −1.12, *P* < 0.00001, *I*^2^ = 94%, Figure 8B).

#### Extravesical ureteral reimplantation vs. intravesical ureteral reimplantation with minimal invasive approach

There are only one study comparing the robotic EVUR with robotic IVUR (15), two studies comparing robotic EVUR with open IVUR (16, 17), and one study comparing laparoscopic EVUR with open IVUR (18). The heterogenicity in the operative approaches does not allow for a meaningful meta-analysis to compare minimal invasive EVUR with minimal invasive IVUR.

#### Extravesical ureteral reimplantation vs. intravesical ureteral reimplantation for bilateral reflux

Six studies reported the incidence of postoperative ARU after bilateral EVUR and IVUR. The bilateral EVUR had significantly higher postoperative ARU (8.1%) compared to bilateral IVUR (1.7%), the pooled OR was 4.40 (95% CI, 1.33–14.58, *P* = 0.02, *I*^2^ = 4%) (Figure 6B). Among the six studies, three reported ARU after open surgery for bilateral VUR. The ARU was also higher in EVUR (5.9%) compared to IVUR (0%). However, it did not reach a statistical difference due to the small sample size (OR = 4.40; 95% CI, 0.49–39.36; *P* = 0.19, *I*^2^ = 0%).

Other outcomes were not available for meta-analysis for bilateral reflux.

## Discussion

Our meta-analysis confirms the prevailing opinion that EVUR and IVUR are equally efficacious in correcting VUR (97.2% for EVUR and 96.7% for IVUR) (8); this is not unexpected since both techniques follow the surgical principle of lengthening the intramural component of the distal ureter. Our study showed that EVUR had shorter LOS and operative time. However, bilateral EVUR was associated with five times increased risk of postoperative ARU than bilateral IVUR.

Acute retention of urine is commonly associated with bilateral EVUR, with reported incidence as high as 26% (10). Most common EVUR techniques are Lich-Gregoir procedure and detrusorrhaphy, a modified Lich-Gregoir technique with ureteral advancement. Both extravesical approaches entails dissection of the involved ureter to the ureteral hiatus and incision of the detrusor muscle of the bladder to create a submucosal tunnel. This dissection near the highly innervated trigone and bladder base may predispose to neuropraxia (23), or even complete disruption of trigonal innervation resulting in postoperative ARU after bilateral EVUR (11). A neuroanatomical study demonstrated the presence of nerve fibers in the medial aspect of the distal ureter which encircle the ureter at the level of the vesicoureteric junction outside Waldeyer’s sheath, suggesting that the only safe area for dissection to avoid nerve injury may be beneath the sheath (24). Hence, nerve injury at different sites can be minimized using either surgical approach – IVUR avoids dissection at the trigone or bladder base, while unilateral EVUR spares the contralateral nerves responsible for voiding. Correspondingly, our study findings showed the absence of ARU episodes in unilateral ureteral reimplantation, and a higher risk of ARU in bilateral EVUR compared to bilateral IVUR (OR 4.40). Most of the ARU (9/11) in the included studies were transient and only required a short-term catheterization. However, one study (11) reported two cases of prolong voiding inefficiency after bilateral EVUR that required vesicostomy and Mitrofanoff procedure. Surgical modifications that minimize the risk of voiding dysfunction in bilateral EVUR include a nerve-sparing technique that has been shown to reduce the incidence of ARU to 2% (25), and a modified Lich-Gregoir technique with limited distal ureter dissection that has been successfully conducted without encountering urinary retention in a series of 50 patients (26).

Intravesical ureteral reimplantation requires cystostomy, and this typically results in postoperative hematuria that lasts on average 2.5–4.2 days (14, 21). This contributes greatly to longer LOS with need for prolonged monitoring, urinary catheterization and/or ureteral stent placement. Duration of hospitalization is also prolonged by the corresponding increased analgesic requirement for postoperative pain and intense bladder spasms (21, 22, 27). Hence the longer LOS associated with IVUR reported in our meta-analysis is likely related to increased postoperative hematuria and postoperative pain related to the operative approach. Proponents of the extravesical technique have reported that it is safe and feasible to perform both bilateral and unilateral open EVUR, using a modified technique, as a day procedure without increased morbidity or rehospitalization (12, 28).

While EVUR may reduce operative time as it does not require a bladder incision, the shorter operative time associated with EVUR found in our study was only for studies on open operations. The three MIS EVUR studies in our meta-analysis that utilized robotic-assisted EVUR (15–17) reported longer operative times compared to open IVUR. Other meta-analyses comparing open EVUR and RALUR have also found increased operative time associated with robotic-assisted EVUR (29, 30).

Ureteral obstruction is a serious potential complication after ureteric reimplantation; hence, it is routine to track postoperative hydronephrosis (or renal pelvic dilatation) as a surrogate marker. Our study did not show a statistically significant difference in terms of postoperative hydronephrosis rates between both surgical approaches. It is possible that the low rate of postoperative hydronephrosis in our meta-analysis and in other pediatric reports may underestimate the stricture complication rate after EVUR (31) since strictures typically develop months later. Selzman et al. reported an 11% stricture rate at 1 year post open ureteral reimplantation for ureteral injury in adults (32–34). However, for pediatric ureteral reimplantation for primary VUR, postoperative hydronephrosis is commonly associated with higher grade VUR, and even if not fully resolved often shows some degree of improvement compared to the extent of preoperative dilatation. Hence in contrast, it may represent residual VUR, transient obstruction from edema or poor ureter peristalsis, rather than permanent obstruction from stricture. In a retrospective study of 938 pediatric ureteral reimplantation patients, 21% had postoperative hydronephrosis, the majority being transient and resolving on average 1.36 years postoperatively, with only 0.1% developing ureteral obstruction (35). This was further validated in a later retrospective review of 25 patients after EVUR where new-onset postoperative hydronephrosis was found to not correlate with reduced postoperative differential renal function (36). Indeed, most of hydronephrosis in our included studies were mild and resolved in few months.

Similar to other surgical operations in this age of MIS, there has been great interest in evolving MIS techniques for ureteral reimplantation (37). Unlike MIS IVUR with novel transvesical pneumobladder techniques, laparoscopic/robotic EVUR, especially the Lich-Gregoir method, was attempted early during the emergence of pediatric reconstructive laparoscopy (38). However, there is very limited comparative study available for a meta-analysis for minimal invasive reimplantations. Babu et al. conducted a meta-analysis using single-arm studies (13 articles of laparoscopic extravesical and 10 articles of trans vesicoscopic ureteric reimplantation) and found that MIS EVUR had higher success, shorter operative time and hospital stay but more post-operative urinary retention compared to MIS IVUR technique (39). Although Babu’s study agrees with our finding, it has to be interpreted carefully as incorporation of single-arm studies in meta-analysis introduces significant patient selection, investigator and institutional over enthusiasm, and publication bias.

Our meta-analysis includes only comparative studies but it also suffers from several limitations. Firstly, our source data is limited to mostly retrospective observational studies that are prone to selection bias and information bias. The only RCT available was considered low quality with Jadad score of 1 star. Ideally more high quality, well-conducted RCTs could confirm the validity of our findings. Nevertheless, given that no other RCTs have been carried out in the past 3 decades, our study represents the best summary of available evidence. The problem of pooled data also resulted in heterogeneity in operative techniques, with MIS predominantly in the extravesical arm. There was also more bilateral reimplantation in IVUR (60.8%) compared to EVUR (34.8%), which could be a bias related to the surgeon’s preference to perform a considered safer procedure for bilateral VUR. Other potential confounders for postoperative VUR persistence were that some studies included children with duplicated collecting system (7 out of 50 children) (16) and voiding dysfunction (3 out of 60 children) (18). Finally, there was insufficient data for meaningful meta-analysis for several other key outcomes of interest like duration of urinary catheterization, and the use of postoperative analgesia and anticholinergic agents.

## Conclusion

Both EVUR and IVUR are equally effective operations for the anatomical correction of persistent high-grade VUR, with no significant difference in the persistence of VUR. EVUR has the advantage of shorter LOS and operative time but is associated with increased risk of postoperative ARU for bilateral operations. More robust prospective RCTs evaluating the peri-operative outcomes of EVUR and IVUR would be required to validate our meta-analysis.

## Data availability statement

The original contributions presented in this study are included in the article/supplementary material, further inquiries can be directed to the corresponding author.

## Author contributions

YC, ZL, UJ, T-LY, CO, SS, AL, YL, and AJ contributed to design, planning, and writing of the manuscript. YC and ZL extracted the data and performed the analysis. ZL, LYO, and YC drafted the initial manuscript which was jointly approved by all authors. YC was the guarantor of the work. All authors contributed to the article and approved the submitted version.

## Abbreviations

VUR: vesicoureteral reflux
EVUR: extravesical vesicoureteral reimplantation
IVUR: intravesical vesicoureteral reimplantation
CI: confidence interval
OR: odds ratio
OCS: observational clinical studies
RCT: randomized controlled trials.
